# Formaldehyde a Cure for Pyorrhea Alveolaris

**Published:** 1913-09-15

**Authors:** G. A. Barnett


### FORMALDEHYDE A CURE FOR PYORRHEA ALVEOLARIS.

After two years of experimenting I have found that a solution of formaldehyde will positively cure pyorrhea in its worst form. You will get almost instant relief where pain is intense, pus will stop flowing within a few days, gums will settle down around necks of teeth, and if the alveola plate is not entirely destroyed, teeth will soon tighten so that if there are any missing teeth they can be replaced with bridgework in a surprisingly short time.

Not like Dr. Friedmann, I will give my chosen profession the benefit of my experience in the treatment of the tubercular condition of the alveoli.

I will report on only one case at this time, though I could give dozens of others, but I consider this particular case as bad as any could be.

Case No. 11.—J. S. W., age about fifty, came to me one year ago, after having been treated for pyorrhea for a number of years. The six inferior anterior teeth were barred together with a cast plate, his gums were greatly inflamed and there was a great abundance of pus around each tooth; a profuse exudation of purulent matter from the pockets, and the patient had not slept for two nights.

I removed what calcareous deposits there was around the necks of the teeth and prescribed ten drops of formaldehyde to half a glass of water, and that if he did not get a slight burning sensation around necks of teeth and under the tongue to keep adding formaldehyde until he did. However, he had to use from twenty to twenty-five drops to half glass of water to get the slight burning sensation which is imperative.

I see this case every sixty days for prophylactic treatment. There are no signs of pus, gums are hard and teeth are tight. Don't let the wise one's think they can put up a preparation of pinked up formaldehyde as a cure for pyorrhea, for the reason that you have to let each individual case test out the amount that his or her gums will stand to get the burning effect.

I usually start them with eight or ten drops to half glass of water and tell them to add to or deduct as necessary; I have one patient that can only use six drops.

A little later I will give what I have found to be the principal cause of pyorrhea.—G. A. Barnett, in *Western Journal.*

